# The correlation between a Th1/Th2 cytokines imbalance and vitamin D level in patients with early chronic obstructive pulmonary disease (COPD), based on screening results

**DOI:** 10.3389/fphys.2023.1032786

**Published:** 2023-03-17

**Authors:** Wenhui Tang, Yan Rong, Hongmei Zhang, Zi’e Zhan, Long Yuan, Yan Ning, Wenji Lin

**Affiliations:** Department of Respiratory & Critical Medicine, Shenzhen Municipal Qianhai Shekou Free Trade Zone Hospital, Shenzhen, China

**Keywords:** acute exacerbation of chronic obstructive pulmonary disease, Th1/Th2, 25-hydroxy-vitamin D, correlation analysis, anti-inflammatory mechanism

## Abstract

**Objective:** This study explored the correlation between a Th1/Th2 cytokines imbalance and 25-hydroxy-vitamin D (vit D) level in early chronic obstructive pulmonary disease (COPD), provided experimental rationales for the role of vit D in the prevention and control of COPD, and elucidated the potential anti-inflammatory mechanism involved.

**Methods:** This study was based on the results of the “Screening and Early Diagnosis of COPD” public health project conducted through Shenzhen Municipal Qianhai Shekou Free Trade Zone Hospital. Patients with early COPD were selected as study participants. A prospective, randomized, and controlled method was employed for assigning eligible participants into three groups, i.e., a COPD lung function (LF) I, COPD LF II, and a healthy group, respectively (n = 40 each). The serum content of tumor necrosis factor alpha (TNF-α), interferon-gamma (IFN-γ), interleukin 4 (IL-4), and IL-6 were measured by enzyme-linked immunosorbent assay, and the ratio of IFN-γ/IL-4 treated as a marker for Th1/Th2. The serum concentration of 25-hydroxyl-vit D (25 [OH]D) was quantified by a chemiluminescence assay. Statistical processing was performed, and the correlations between changes in the above parameters with vit D level and LF parameters were examined.

**Results:** There were differences in FEV1pred%, FEV1/FVC, IFN-γ, IL-4, IL-6 and IFN-γ/IL-4 between the healthy group, the COPD LF I group and the COPD LF II group (*p* < 0.05). In early COPD, Th1/Th2 cytokines was positively correlated with forced expiratory volume/expected value (FEV1pred%) (r = 0.485, *p* < 0.001) and forced expiratory volume/forced vital capacity (FEV1/FVC) (r = 0.273, *p* = 0.018); Th1/Th2 cytokines levels positively correlated with vit D level (r = 0.27, *p* = 0.02), and 25(OH)D level positively correlated with FEV1pred% (r = 0.695, *p* < 0.001).

**Conclusion:** Vitamin D deficiency was ubiquitous in patients with early COPD. It was positively correlated with the FEV1pred% and FEV1/FVC LF parameters. Accordingly, this study provides experimental rationales for the role of vit D in the prevention and control of COPD and the potential anti-inflammatory mechanisms involved.

## 1 Introduction

With characteristics that include respiratory system symptoms and limited airflow, (Goldcopd, 2021) chronic obstructive pulmonary disease (COPD) is currently the third-highest cause of mortality worldwide (Labaki and Rosenberg, 2020). The disturbance of T-helper-cell (Th) subsets (Th1/Th2) has been known to play an important role in the pathogenesis of COPD (Zhang et al., 2013). Some studies have shown that in addition to the abnormal airway anatomy, airflow restriction and severe pulmonary dysfunction, patients with COPD also have an imbalance of immune function (Valero et al., 2017; Firacative et al., 2018). Th1 cells mainly mediate cellular immune response. Th2 cells mainly participate in humoral immunity and are closely related to allergic diseases. Interferon-gamma (IFN-γ) and Interleukin-4 (IL-4) are typical cytokines secreted by Th1 and Th2 cells, respectively. In several studies (Zhang et al., 2013), the ratio of IFN-γ/IL-4 has been shown to represent a superior Th1/Th2 status. Moreover, Th1 and Th2 cells also secrete other cytokines, such as TNF-α and IL-6, which were also used to reflect the status of Th1 and Th2 cells. Therefore, Th1/Th2 cytokines imbalance is closely related to the occurrence and development of infection and inflammatory diseases such as COPD.

Vitamin D (vit D) is an essential vitamin for human body and an important immune regulatory factor, which can affect the expression of related immune factors in patients with COPD. In addition to regulating the homeostasis of calcium, phosphate, and bone mineralization, 25-hydroxy-vitamin D (25 [OH]D) also influences the level of airway inflammatory factors in COPD by directly or indirectly acting on Th cells (Celli et al., 2008). A 25(OH)D serum level below 20 ng/mL (50 nmol/L) was defined as a vit D deficiency (VDD) (Zhang et al., 2010). Current studies have detected a correlation between COPD and VDD (Monadi et al., 2012; Burkes et al., 2020). These studies found that the level of vit D in patients with COPD was positively correlated with pulmonary function. The lower the vit D is, the worse the lung function is, indicating that the vit D level can reflect the degree of lung function damage to a certain extent.

According to the Global Initiative for Chronic Obstructive Lung Disease, grade I/II COPD is part of the early phase of the disease. The total number of patients with early COPD is extremely high (Zhou et al., 2017). In COPD cases, lung function (LF) can decline rapidly, and patients are rarely diagnosed in a timely manner (Vestbo et al., 2011). Therefore, early detection and prompt treatment management remain essential for the prevention and control of COPD (Heulens et al., 2015).

Based on the above rationales, the hospital department of the current authors undertook the “Screening and Early Diagnosing of COPD” public health project. Based on the screening results for COPD, a correlational study was conducted focusing on a Th1/Th2 cytokines imbalance and vit D level in patients with early COPD. Changes in Th1/Th2 imbalance and vit D expression level were detected and their correlations with LF parameters were examined. As a result, this study offers experimental rationales for the role of vit D in the prevention and control of COPD and elucidates the potential anti-inflammatory mechanisms involved.

## 2 Data and method

### 2.1 Study participants

A public health project, “Screening and Early Diagnosing of COPD,” was undertaken in the Department of Respiratory and Critical Medicine of Shenzhen Municipal Qianhai Shekou Free Trade Zone Hospital. COPD screening questionnaires (COPD-SQs) was based on the Burden of Obstructive Lung Disease (BOLD) study and the 2007 version of the Chinese Epidemiological Questionnaire (Zhong et al., 2007; Kulbacka-Ortiz et al., 2022). The risk factors in COPD-SQs included age, smoking package years, body mass index, chronic cough, shortness of breath, whether to use biofuel or coal for cooking or heating, and whether parents, brothers and sisters had chronic bronchitis, emphysema, asthma, and COPD history. Using an online survey platform, a total of 3,800 COPD-SQs were collected from local permanent residents aged 20 years and older, including 423 high-risk COPD individuals. Routine LF examinations were performed and, according to the diagnostic criteria of the 2022 *Global Strategies for Diagnosing, Managing and Preventing Early COPD*, (Goldcopd, 2021) LF grades I/II (defined by a Forced expiratory volume at the first second/forced vital capacity (FEV1/FVC) of <0.7, and a forced expiratory volume/expected value (FEV1 [pred%]) of ≥50% after inhaling through a bronchodilator; negative bronchial dilation test). Specifically, FVC was the maximum volume of air that can be exhaled as soon as possible after trying to inhale as much as possible. FEV1 was the volume of exhaled volume in the first second after the maximum deep inspiration. FEV1/FVC was defined as the ratio of FEV1 value and FVC value. FEV1 [pred%]) was defined as the ratio measured FEV1 value and calculated FEV1 value. Negative bronchial dilation test was defined as that after inhalation of bronchodilator, the improvement rate of forced expiratory volume in the first second of the patient was less than 12%, and the increase of absolute value was less than 200 mL. In addition, two experienced physicians made a differential diagnosis of suspected asthma and excluded asthmatics. Finally, a total of 193 patients with early COPD was confirmed and selected as the study participants. Based on a sequential presentation order, a prospective randomized controlled method was employed for assigning eligible subjects into three groups, i.e., the COPD LF I and COPD LF II groups, as well as a healthy group (n = 40 each). The healthy group included individuals from the health examination center of the authors’ hospital. These cases had no symptoms and medical history of CODP, and X-ray examination and pulmonary function examination were normal. Among the three groups, no marked differences were observed in age, gender, smoking history, or body mass index (BMI). Additionally, all participants had normal liver/kidney function and did not use medications interfering with conversion/absorption of vit D.

This study was conducted with approval from the Ethics Committee of Shenzhen Municipal Qianhai Shekou Free Trade Zone Hospital (No. 2022-107). This study was conducted in accordance with the declaration of Helsinki. Written informed consent was obtained from all participants.

### 2.2 Study methods

#### 2.2.1 Data collection

a) The general patient profiles included age, gender, smoking history, and BMI;
b) Lung function: An LF analyzer (GUARK PET3) was used for collecting samples that would be used for analyzing the first second of FEV1pred%, the first second of forced expiratory volume/forced vital capacity (FEV1/FVC), and the bronchial dilation test.
c) Measuring Th1/Th2 cytokines: Peripheral blood was collected from the study subjects in 3–5 mL, naturally clotted at room temperature for 15 min, then centrifuged at 2000-4,000 r/min for 20 min, and 0.5 mL of the separated serum was taken out and assayed within 4 h using a flow cytometer (Jiangxi Saiki Biotechnology Co., Ltd.). TNF-α, IFN-γ, IL-4, IL-6 were collected and IFN-γ/IL-4 ratio was calculated.
d) The detection of serum 25(OH)D level: A 25(OH)D reagent kit (the chemiluminescence method) was purchased from Shenzhen New Industry Biomedical Engineering Shareholding Ltd. The procedures were performed following the kit instructions.

#### 2.2.2 Statistical processing

The SPSS Statistics 26.0 software program was used to conduct statistical processing. Quantitative data conforming to normal distribution were expressed as mean ± standard error (x̄ ± s); the data that did not conform to a normal distribution were expressed as the median (quartile). In the three groups, analysis of variance was used for quantitative data that conformed to normal distribution. Variations were analyzed using inter-group paired comparisons, for which both a *t*-test and a rank-sum test were applied; *p* < 0.05 was adopted as a threshold value for judging statistical difference. The Pearson’s/Spearman’s correlation method was employed for examining the correlations between the serum levels of 25(OH)D, TNF-α, IFN-γ, IL-4, IL-6, and LF parameters. *p* < 0.05 was considered to indicate a statistically significant difference.

## 3 Results

### 3.1 Comparison of baseline information among the three groups

The results revealed that there were no statistical differences in sex and BMI among the COPD LF I, COPD LF II, and healthy groups (*p* > 0.05) (Table 1).

**TABLE 1:** Comparison of baseline information among the three groups.

Items	Healthy group (n = 40)	COPD LF I (n = 40)	COPD LF II (n = 40)	*p*-value
Gender				0.524
Male	27 (67.5%)	27 (67.5%)	31 (77.5%)	
Female	13 (32.5%)	13 (32.5%)	9 (22.5%)	
Age (years)				<0.001
20–49	23 (57.5%)	15 (37.5%)	13 (32.5%)	
50–59	15 (37.5%)	12 (30.0%)	6 (15.0%)	
60–69	2 (5.0%)	7 (17.5%)	7 (17.5%)	
≥70	0 (0.0%)	6 (15.0%)	14 (35.0%)	
Smoking Quantity (branch/day)				0.030
0–14	29 (72.5%)	20 (50.0%)	20 (50.0%)	
15–30	7 (17.5%)	7 (17.5%)	7 (17.5%)	
≥30	4 (10.0%)	13 (32.5%)	13 (32.5%)	
BMI				0.894
<18.5	3 (7.5%)	9 (22.5%)	6 (15.0%)	
18.5–23.9	26 (65.0%)	17 (42.5%)	20 (50.0%)	
24.0–27.9	6 (15.0%)	10 (25.0%)	11 (27.5%)	
≥28	5 (12.5%)	4 (10.0%)	3 (7.5%)	

Lung function, LF; chronic obstructive pulmonary disease, COPD; body mass index, BMI.

### 3.2 Comparison of lung function-related indexes, 25(OH)D levels, cytokines, and Th1/Th2 among the three groups

FEV1pred%, FEV1/FVC, and 25(OH)D were higher to lower in the healthy group > the COPD LF I group > the COPD LF II group (all *p* < 0.001). The levels of TNF-α, IFN-γ, IL-4 and IL-6 in the COPD LF II group were higher than those in the COPD LF I group and the healthy group (all *p* < 0.001). Th1/Th2 (IFN-γ/IL-4) in the COPD LF II group was lower than that in the COPD LF I group but higher than that in the healthy group (*p* < 0.05) (Table 2).

**TABLE 2:** Comparison of lung function-related indexes, 25(OH)D levels, cytokines, and Th1/Th2 among the three groups.

Items	Healthy group (n = 40)	COPD LF I (n = 40)	COPD LF II (n = 40)	*p*-value
FEV1pred%	98.12 (91.30, 107.09)	85.72 (81.09, 89.48) ^▲^	58.52 (54.23, 68.51) ^▲★^	<0.001
FEV1/FVC	81.27 (78.23, 91.07)	65.23 (57.96, 67.08) ^▲^	64.21 (57.12, 68.22) ^▲^	<0.001
25(OH)D (ng/mL)	22.80 (2.32)	18.19 (1.71) ^▲^	15.10 (1.45) ^▲★^	<0.001
TNF-α(pg/ml)	1.05 (0.98, 1.34)	1.51 (1.44, 1.55)	1.57 (1.23, 1.81)^▲^	<0.001
IFN-γ (pg/mL)	1.18 (1.01, 1.25)	1.55 (1.31, 1.78) ^▲^	1.76 (1.71, 2.10) ^▲★^	<0.001
IL-4 (pg/mL)	1.20 (1.02, 1.77)	1.05 (0.98, 1.34) ^▲^	1.57 (1.50, 1.78) ^▲★^	<0.001
IL-6 (pg/mL)	2.20 (1.81, 2.26)	1.02 (0.81, 7.77) ^▲^	22.66 (21.83, 23.63) ^▲★^	<0.001
IFN-γ/IL-4	0.92 (0.69, 1.22)	1.34 (1.16, 1.61) ^▲^	1.15 (1.01, 1.32) ^▲★^	<0.001

Compared with the healthy group.

▲*p* < 0.05; compared with COPD LF I.

★*p* < 0.05; Lung function, LF; chronic obstructive pulmonary disease, COPD; forced expiratory volume in the first second, FEV1; Forced vital capacity, FVC; Interferon-gamma, IFN; tumor necrosis factor alpha, TNF; interleukin, IL.

### 3.3 The correlation between Th1/Th2 cytokines and LF of early COPD

**FIGURE 1:**
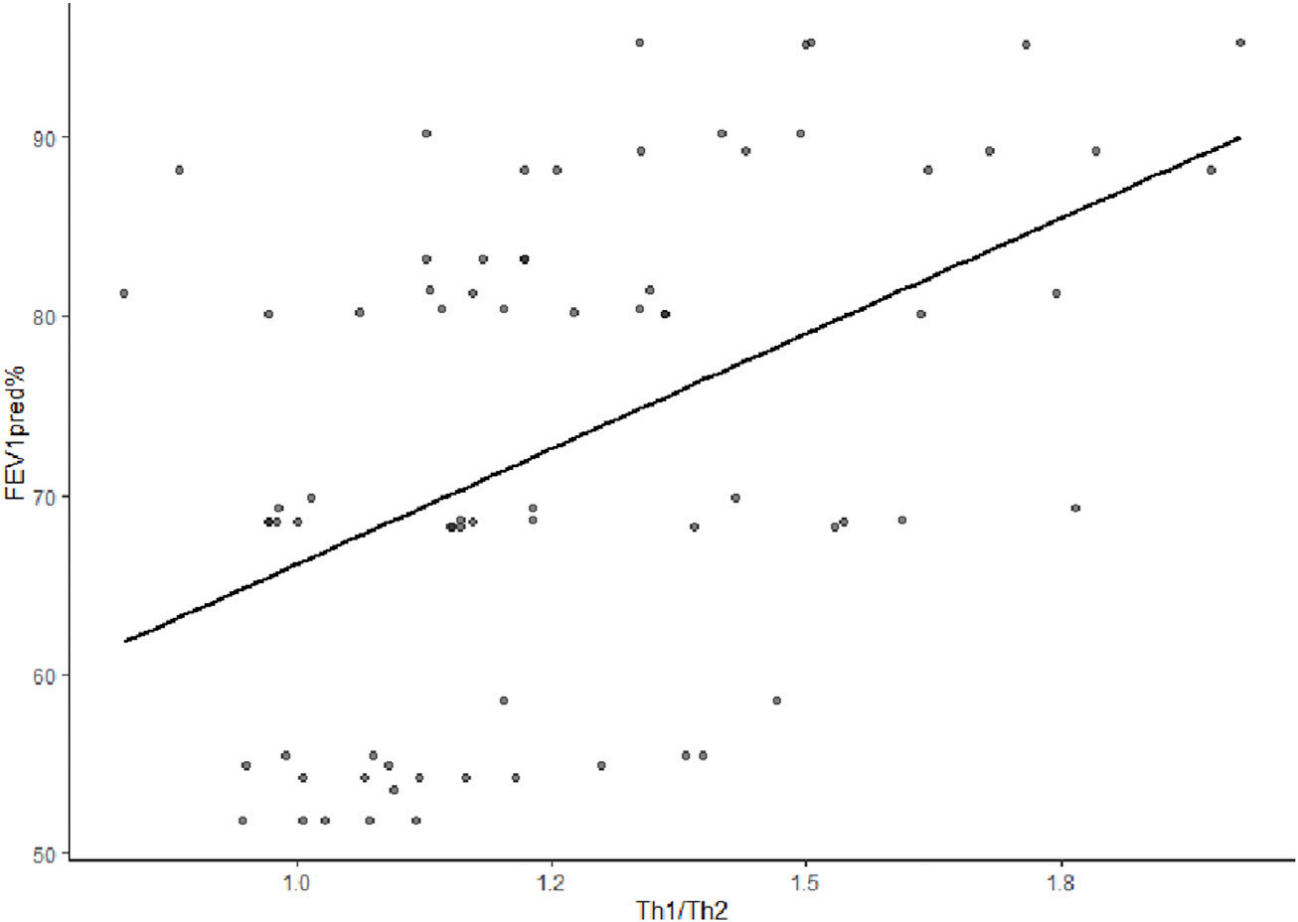
Correlation analysis between Th1/Th2 cytokines and FEV1pred% in early COPD. The r (correlation coefficient) was 0.485(*p* < 0.001), indicating a positive correlation between Th1/Th2 cytokines and FEV1pred%.

**FIGURE 2:**
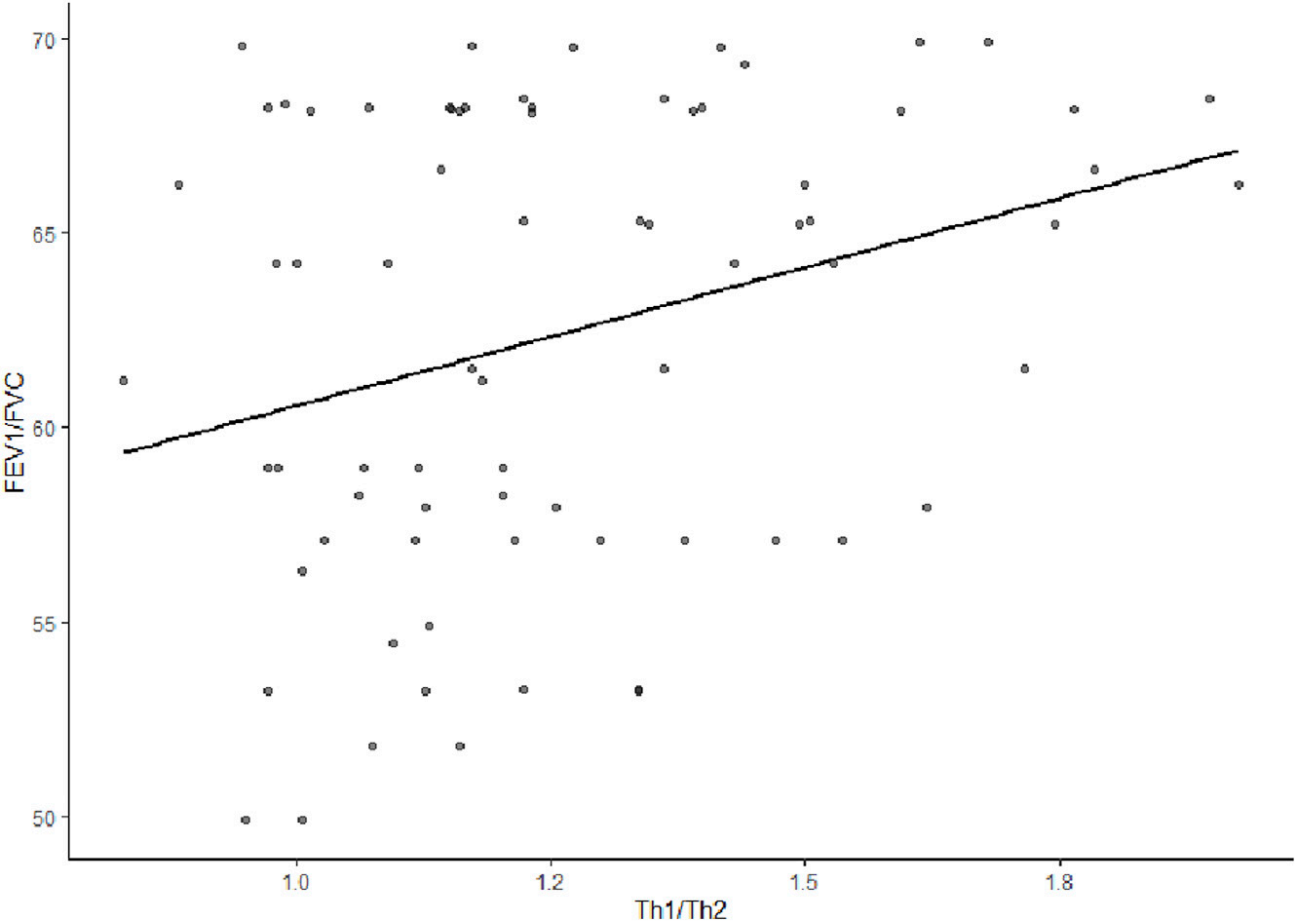
Correlation analysis between Th1/Th2 cytokines and FEV1/FVC in early COPD. The r (correlation coefficient) was 0.273(*p* = 0.018), indicating a positive correlation between Th1/Th2 cytokines and FEV1/FVC.

Th1/Th2 cytokines was positively correlated with FEV1pred% and FEV1/FVC of early COPD, with correlation coefficients of r = 0.485 (*p* < 0.001) and r = 0.273 (*p* = 0.018), respectively (Figure 1; Figure 2).

### 3.4 The correlation analysis between Th1/Th2 cytokines and vitamin D-level in early COPD

**FIGURE 3:**
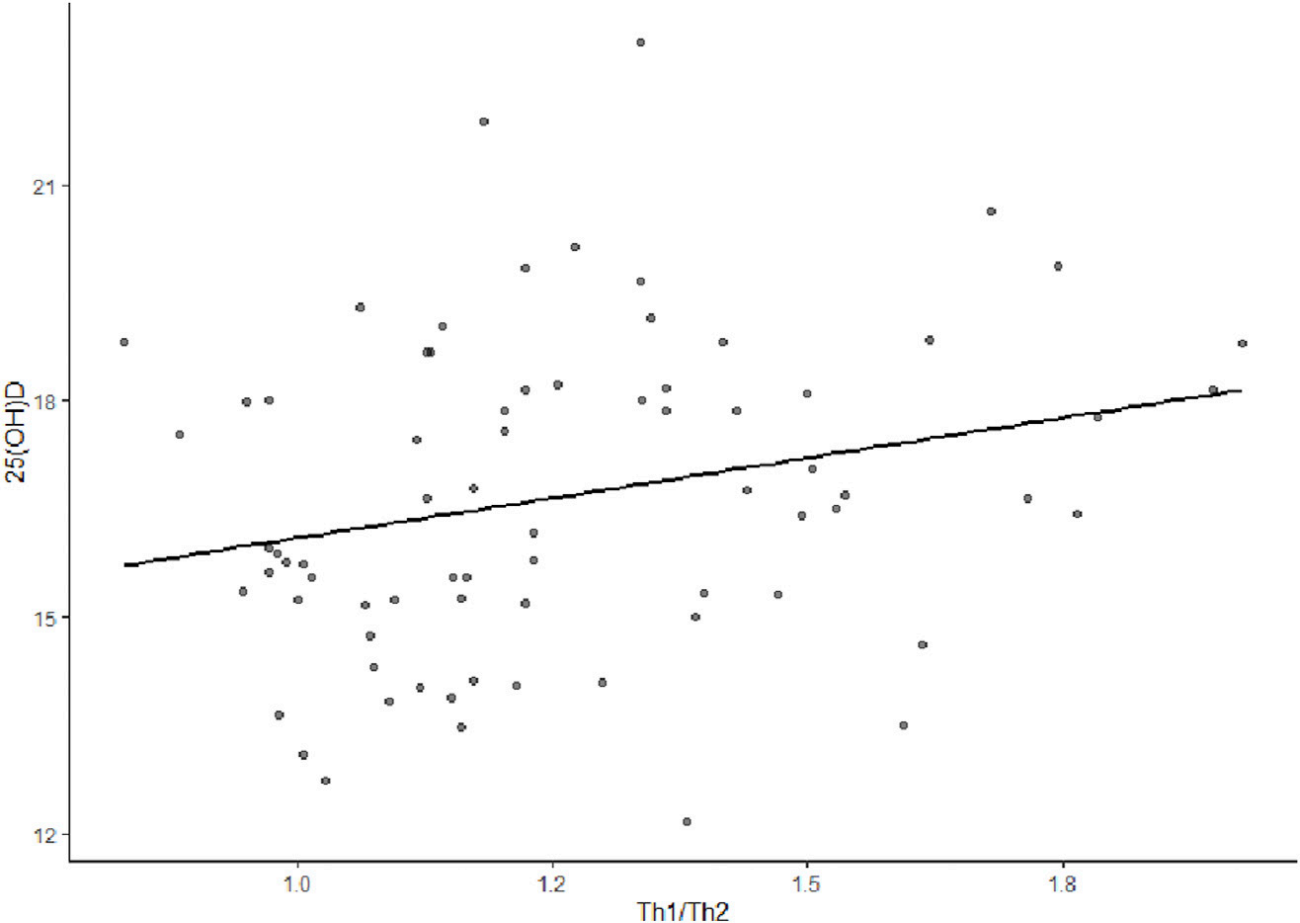
Correlation analysis between Th1/Th2 and vit D level in early COPD. The r (correlation coefficient) was 0.27 (*p* = 0.02), indicating that Th1/Th2 and vit D were positively correlated.

It was showed a positive correlation between Th1/Th2 cytokines and vitamin D-level in early COPD, with the correlation coefficient of r = 0.27(*p* = 0.02) (Figure 3).

### 3.5 Correlation analysis between the serum concentration of 25-hydroxyl-vit D level and LF in early COPD

**FIGURE 4:**
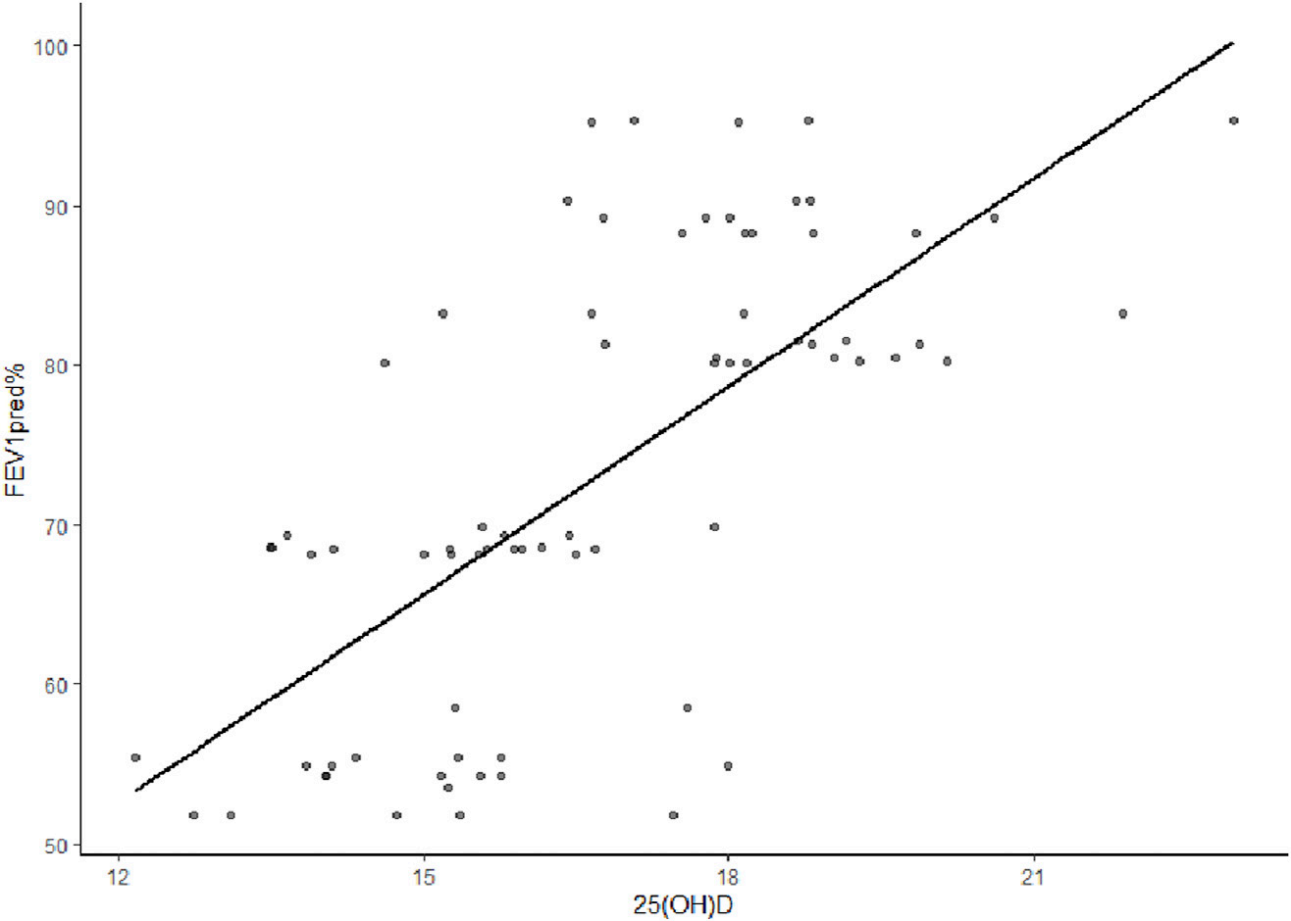
Correlation analysis between 25(OH)D level and FEV1pred% in early COPD. The 25(OH)D level and FEV1/FVC were positively correlated, with a r (correlation coefficient) of 0.695 (*p* < 0.001).

The 25(OH)D levels were positively correlated with FEV1pred% in early COPD, with a correlation coefficient of r = 0.695 (*p* < 0.001). However, the 25(OH)D levels were not correlated with FEV1/FVC (*p* > 0.05) (Figure 4).

## 4 Discussion

The major pathogenic factors and mechanisms of COPD include chronic inflammatory responses, an imbalance in protease/anti-protease, oxidation stress, and epithelial apoptosis (Zhong et al., 2007). Pulmonary and systemic inflammations were two additional important pathological causes (Group of Chronic Obstructive Pulmonary Disease et al., 2021; Wei et al., 2021). As a core inducer of progressive COPD, (Vestbo et al., 2011) inflammation reflects a disease course in which multiple inflammatory cells and mediators participate. A T-cell-induced immune disturbance was closely correlated with the occurrence and progression of COPD (Singh et al., 2018). Furthermore, Th1 could produce IL-2, TNF-α, and IFN-γ to induce T-cell proliferation, boost the *in vivo* killing activities of related effector cells, and facilitate the expression and release of pro-inflammatory mediators, causing tissue injury and reconstruction (Singh et al., 2018). Moreover, Th2 cells may cause pathological changes by secreting IL-4 and IL-6, stimulating B-cells to secrete immunoglobulin E, and inducing airway epithelial cells to express eotaxin, which promotes eosinophilic inflammation (Muhammad Yusoff et al., 2020). The typical pathological changes of COPD included sustained inflammation, emphysema, and airway reconstructing (Wei and Li, 2018).

Multiple immune modulation imbalances play an important role in the development and progression of chronic obstructive pulmonary disease. In this study, the levels of TNF-α, IFN-γ, IL-4, and IL-6 were all higher in the COPD LF II group than in the COPD LF I and healthy groups. However, the IFN-γ/IL-4 ratio was lower, which was supported by the results in a study conducted by Chen et al. (Chen et al., 2020) According to some research, compared with healthy controls, both Th1 cells and IFN-γ were elevated in patients with early COPD. IFN-γ suppressed not only the function of Th2 cells but also activated neutrophils and stimulated macrophages to participate in the airway inflammation of COPD (Egli et al., 2018). In addition, IFN-γ also promotes the expression of interferon by promoting expression of interferon-inducible protein 10 and monocyte factor, thereby promoting lung macrophages to secrete matrix metallo-proteinase-12 (MMP-12), which further causes lung tissue destruction and advancing emphysema formation (Li et al., 2021). Both Th2 cells and IL-4 increase in patients with early COPD. A high expression of IL-4 could also promote the differentiation of Th2 cells and suppress the production of Th1. IL-4 can disrupt normal airway defense mechanisms by promoting macrophage activation and proliferation of fibroblasts and endothelial cells, increasing inflammatory cell recruitment to the lung (Wei and Li, 2018).

Chronic obstructive pulmonary disease is characterized by the limitation of airflow. As revealed in the present study, the Th1/Th2 cytokines in early COPD may be positively correlated with FEV1pred% and FEV1/FVC. The current findings agreed with the research results of Silva et al. (Silva et al., 2018) A possible mechanism involved in this finding was that a release of pulmonary inflammatory mediators from COPD inflammation-originating sites brought about changes in airway cells, greater sedimentation of airway collagen fibers, airway reconstruction, and lower LF. Some studies have indicated that IFN-γ could promote the release of porin and granzyme B from cytotoxic T-cells, which was related to the apoptosis of bronchial epithelial cells and caused the breakdown of pulmonary structural cells and the degradation and reconstruction of the extracellular matrix, leading to the obstruction of small airways (Williams et al., 2021). Based on its capability to promote the secretion of transforming growth factor beta from eosinophil, IL-4 was linked to airway fibrosis (Barnes, 2016).

In the present study, the most critical finding showed that 25(OH)D in the COPD LF I group and the COPD LF II group was lower than that in the healthy group. The phenomenon of VDD is generally ubiquitous in early COPD. In the present study, almost 100% of patients with early COPD had VDD. To date, several studies have demonstrated that VDD was negatively correlated with LF and disease severity in patients with COPD (Monadi et al., 2012). Vit D has the functions of anti inflammation, regulating immunity and improving airway remodeling. It was reported that 1,25-dihydroxyvitamin D3 regulated inflammation through multiple signal transduction pathways in macrophages and airway epithelial cells, reducing lung tissue damage and slowing down the progression of COPD (Xiong et al., 2017). In addition, some studies also demonstrated that Vit D inhibited T lymphocytes from secreting pro-inflammatory factors such as IL-17 and IL-6, inhibiting Th17 cell immune response and controlling inflammatory response of diseases (Gu et al., 2015). In the report of global initiative for chronic obstructive lung disease (GOLD) in 2022, it was recommended that all patients hospitalized for severe vitamin D deficiency to be evaluated and investigated to determine if they are severely deficient (<10 μg/L or <25 nmol/L) and supplementation if needed (GOLD Reports, 2022). However, whether or not vit D status may impact the occurrence and progression of COPD remains unclear. The evidence for the role of vit D in immune regulation and protection has been derived from observational studies. There is no definite conclusion on the correlations between vit D, various clinical inflammatory parameters, and pulmonary ventilation function. The present study also indicated a positive correlation between Th1/Th2 and vit D levels in early COPD (r = 0.27, *p* = 0.02). Additionally, the 25(OH)D level positively correlated with FEV1pred% in early COPD (r = 0.695, *p* < 0.001). These results validate the previous finding that Th1/Th2 is positively correlated with FEV1pred%.

In conclusion, VDD was found to be ubiquitous in patients with early COPD and positively correlated with FEV1pred% and FEV1/FVC. Accordingly, this study offers experimental rationales for the role of vit D in the prevention and control of COPD and the potential anti-inflammatory mechanisms involved.

## Data Availability

The original contributions presented in the study are included in the article/supplementary material, further inquiries can be directed to the corresponding author.

## References

[B1] Barnes P. J. (2016). Inflammatory mechanisms in patients with chronic obstructive pulmonary disease. J. Allergy Clin. Immunol. 138 (1), 16–27. 10.1016/j.jaci.2016.05.011 27373322

[B2] Burkes R. M. Ceppe A. S. Doerschuk C. M. Couper D. Hoffman E. A. Comellas A. P. (2020). Associations between 25-hydroxy-vitamin D levels, lung function, and exacerbation outcomes in COPD: An analysis of the SPIROMICS cohort. Chest 157, 856–865. 10.1016/j.chest.2019.11.047 31958447 PMC7118244

[B3] Celli B. R. Thomas N. E. Anderson J. A. Ferguson G. T. Jenkins C. R. Jones P. W. (2008). Effect of pharmacotherapy on rate of decline of lung function in chronic obstructive pulmonary disease: Results from the TORCH study. Am. J. Respir. Crit. Care Med. 178 (4), 332–338. 10.1164/rccm.200712-1869OC 18511702

[B4] Chen J. Li X. Huang C. Lin Y. Dai Q. (2020). Change of serum inflammatory cytokines levels in patients with chronic obstructive pulmonary disease, pneumonia and lung cancer. Technol. Cancer Res. Treat. 19, 1533033820951807. 10.1177/1533033820951807 33111646 PMC7607805

[B5] Egli A. Mandal J. Schumann D. M. Roth M. Thomas B. Lorne Tyrrell D. (2018). IFNΛ3/4 locus polymorphisms and IFNΛ3 circulating levels are associated with COPD severity and outcomes. BMC Pulm. Med. 18 (1), 51. 10.1186/s12890-018-0616-6 29562888 PMC5861655

[B6] Firacative C. Gressler A. E. Schubert K. Schulze B. Muller U. Brombacher F. (2018). Identification of T helper (Th) 1- and Th2-associated antigens of Cryptococcus neoformansin a murinemodel of pulmonary infection. Sci. Rep. 8 (1), 2681. 10.1038/s41598-018-21039-z 29422616 PMC5805727

[B7] GOLD Reports (2022). Global strategy for the prevention,diagnosis and management of COPD:2022 report[EB/OL]. (2021-11-15)[2022-04-06]. https://goldcopd.org/2022-gold-reports-2.

[B8] Goldcopd (2021). Global strategy for diagnosis, management and prevention of COPD 2022 update (2022 report). [EB/OL], [2021-11-15]. Available at: https://goldcopd.org/goldreports/.

[B9] Group of Chronic Obstructive Pulmonary Disease, Branch of Respiratory Diseases, Chinese Medical Association (2021). Guidelines for the diagnosis and management of chronic obstructive pulmonary disease (revised version 2021). Chin. J. Tuberc. Respir. Dis. 44 (3), 170–205. 10.3760/cma.j.cn112147-20210109-00031 33721932

[B10] Gu Lei Xu Qing Cao Hui (2015). Research progress on the regulation of Th17 cells by 1,25-dihydroxyvitamin D3. J. Pract. Med. 31 (24), 4141–4142.

[B11] Heulens N. Korf H. Cielen N. De Smidt E. Maes K. Gysemans C. (2015). Vitamin D deficiency exacerbates COPD-like characteristics in the lungs of cigarette smoke-exposed mice. Respir. Res. 16, 110. 10.1186/s12931-015-0271-x 26376849 PMC4574263

[B12] Kulbacka-Ortiz K. Triest F. J. J. Franssen F. M. E. Wouters E. F. M. Studnicka M. Vollmer W. M. (2022). Restricted spirometry and cardiometabolic comorbidities: Results from the international population based BOLD study. Respir. Res. 23 (1), 34. 10.1186/s12931-022-01939-5 35177082 PMC8855577

[B13] Labaki W. W. Rosenberg S. R. (2020). Chronic obstructive pulmonary disease. Ann. intern Med. 73 (3), ITC17–ITC32. 10.7326/AITC202008040 32745458

[B14] Li M. H. Marty-Santos L. M. van Ginkel P. R. McDermott A. E. Rasky A. J. Lukacs N. W. (2021). The lung elastin matrix undergoes rapid degradation upon adult loss of hox5 function. Front. Cell Dev. Biol. 9, 767454. 10.3389/fcell.2021.767454 34901011 PMC8662386

[B15] Monadi M. Heidari B. Asgharpour M. Firouzjahi A. Monadi M. Ghazi Mirsaied M. A. (2012). Relationship between serum vitamin D and forced expiratory volume in patients with chronic obstructive pulmonary disease (COPD). Casp. J. Intern Med. 3 (3), 451–455. PMC3755852 24009913

[B16] Muhammad Yusoff F. Wong K. K. Mohd Redzwan N. (2020). Th1, Th2 and Th17 cytokines in systemic lupus erythematosus. Autoimmunity 53 (1), 8–20. 10.1080/08916934.2019.1693545 31771364

[B17] Silva B. S. A. Lira F. S. Ramos D. Uzeloto J. S. Rossi F. E. Freire A. P. C. F. (2018). Severity of COPD and its relationship with IL-10. Cytokine 106, 95–100. 10.1016/j.cyto.2017.10.018 29108795

[B18] Singh S. Verma S. K. Kumar S. Ahmad M. K. Nischal A. Singh S. K. (2018). Correlation of severity of chronic obstructive pulmonary disease with potential biomarkers. Immunol. Lett. 196, 1–10. 10.1016/j.imlet.2018.01.004 29329680

[B19] Valero M. A. Perez-Crespo I. Chillón-Marinas C. Khoubbane M. Quesada C. Reguera-Gomez M. (2017). Fasciola hepatica reinfection potentiates a mixed Th1/Th2/Th17/Treg response and correlates with the clinical phenotypes of anemia. Plos One 12, e0173456. 10.1371/journal.pone.0173456 28362822 PMC5376296

[B20] Vestbo J. Edwards L. D. Scanlon P. D. Yates J. C. Agusti A. Bakke P. (2011). Changes in forced expiratory volume in 1 second over time in COPD. N. Engl. J. Med. 365 (13), 1184–1192. 10.1056/NEJMoa1105482 21991892

[B21] Wei B. C. Li S. (2018). Changes in Th1/Th2-producing cytokines during acute exacerbation chronic obstructive pulmonary disease. J. Int. Med. Res. 46 (9), 3890–3902. 10.1177/0300060518781642 29950127 PMC6136028

[B22] Wei W. L. Wu S. F. Li Z. W. Li H. J. Qu H. Yao C. L. (2021). Exploration of bioactive constituents and immunoregulatory mechanisms of a hanshi-yufei formulation for treating COVID-19. World J. Tradit. Chin. Med. 7, 339–346. 10.4103/wjtcm.wjtcm_45_21

[B23] Williams M. Todd I. Fairclough L. C. (2021). The role of CD8+ T lymphocytes in chronic obstructive pulmonary disease: A systematic review. Inflamm. Res. 70 (1), 11–18. 10.1007/s00011-020-01408-z 33037881 PMC7806561

[B24] Xiong F. Dai H. T. Luo G. (2017). Study on the correlation between serum magnesium, calcium and vitamin D levels and quality of life scores in patients with stable COPD. Zhejiang Med. 39 (17), 1445–1448.

[B25] Zhang F. Q. Zheng J. P. Wang J. H. Lu W. B. Wu R. X. Li X. S. (2010). Comparison of lung volume response with airflow response to bronchodilator in patients with chronic obstructive pulmonary disease. Chin. J. Tuberc. Respir. Dis. 33 (2), 109–113. 20367950

[B26] Zhang R. B. Tan X. Y. He Q. Y. (2013). Current diagnostic shortfalls of chronic obstructive pulmonary disease in mainland China as viewed from the results of epidemiological surveys. Chin. J. Health Manag. 7 (1), 44–47.

[B27] Zhong N. Wang C. Yao W. Chen P. Kang J. Huang S. (2007). Prevalence of chronic obstructive pulmonary disease in China: A large, population-based survey. Am. J. Respi Crit. Care Med. 176 (8), 753–760. 10.1164/rccm.200612-1749OC 17575095

[B28] Zhou Y. Zhong N. S. Li X. Chen S. Zheng J. Zhao D. (2017). Tiotropium in early-stage chronic obstructive pulmonary disease. N. Engl. J. Med. 377 (10), 923–935. 10.1056/NEJMoa1700228 28877027

